# Efficacy of a brief manualized intervention Managing Cancer and Living Meaningfully (CALM) adapted to German cancer care settings: study protocol for a randomized controlled trial

**DOI:** 10.1186/s12885-015-1589-y

**Published:** 2015-08-19

**Authors:** Katharina Scheffold, Rebecca Philipp, Dorit Engelmann, Frank Schulz-Kindermann, Christina Rosenberger, Karin Oechsle, Martin Härter, Karl Wegscheider, Florian Lordick, Chris Lo, Sarah Hales, Gary Rodin, Anja Mehnert

**Affiliations:** 1 Department and Outpatient Clinic of Medical Psychology, University Medical Center Hamburg-Eppendorf, Martinistrasse 52, Hamburg, 20246 Germany; 2 Department of Medical Psychology and Medical Sociology, Section of Psychosocial Oncology, University Medical Center Leipzig, Philipp-Rosenthal-Strasse 55, Leipzig, 04103 Germany; 3 Department of Oncology, Hematology and Bone Marrow Transplantation with section of Pneumology, University Medical Center Hamburg-Eppendorf, Hamburg, Germany; 4 Department of Medical Biometry and Epidemiology, University Medical Center Hamburg-Eppendorf, Martinistrasse 52, Hamburg, 20246 Germany; 5 University Medical Center Leipzig, University Cancer Center Leipzig (UCCL), Liebigstrasse 20, Leipzig, 04103 Germany; 6 Department of Supportive Care, 16-724, Princess Margaret Cancer Centre, 610 University Avenue, Toronto, ON M5G 2M9 Canada

## Abstract

**Background:**

Although psycho-oncological interventions have been shown to significantly reduce symptoms of anxiety and depression and enhance quality of life, a substantial number of patients with advanced cancer do not receive psycho-oncological interventions tailored to their individual situation. Given the lack of reliable data on the efficacy of psycho-oncological interventions in palliative care settings, we aim to examine the efficacy of a brief, manualized individual psychotherapy for patients with advanced cancer: Managing Cancer and Living Meaningfully (CALM). CALM aims to reduce depression and death anxiety, to strengthen communication with health care providers, and to enhance hope and meaning in life. We adapted the intervention for German cancer care settings.

**Methods/Design:**

We use a single-blinded randomized-controlled trial design with two treatment conditions: intervention group (IG, CALM) and control group (CG). Patients in the CG receive a usual non-manualized supportive psycho-oncological intervention (SPI). Patients are randomized between the IG and CG and assessed at baseline (t_0_), after three (t_1_) and after 6 months (t_2_). We include patients with a malignant solid tumor who have tumor stages of III or IV (UICC classification). Patients who are included in the study are at least 18 years old, speak German fluently, score greater than or equal to nine on the PHQ-9 or/and greater than or equal to five on the Distress Thermometer. It is further necessary that there is no evidence of severe cognitive impairments. We measure depression, anxiety, distress, quality of life, demoralization, symptom distress, fatigue as well as spiritual well-being, posttraumatic growth and close relationship experiences using validated questionnaires. We hypothesize that patients in the IG will show a significantly lower level of depression 6 months after baseline compared to patients in the CG. We further hypothesize a significant reduction in anxiety and fatigue as well as significant improvements in psychological and spiritual well-being, meaning and post-traumatic growth in the IG compared to CG 6 months after baseline.

**Discussion:**

Our study will contribute important statistical evidence on whether CALM can reduce depression and existential distress in a German sample of advanced and highly distressed cancer patients.

**Trial registration:**

ClinicalTrials.gov NCT02051660

## Background

Worldwide, eight million people died from cancer in 2010 - an increase of 38 % over the last two decades [1]. Numerous studies have shown high levels of symptom burden and psychosocial distress associated with advanced cancer [2–6]. Psychological distress can range from normal adaptive emotions through to higher levels of severe and clinically significant symptoms that fulfill the standardized diagnostic criteria for adjustment disorder, anxiety disorders, or depression [7]. Studies demonstrate a total prevalence for any mental disorder of 32 % across all tumor settings and stages [8] and prevalence rates of 30–50 % for all mood disturbances including adjustment disorders and depression in palliative care settings [9, 10].

Many patients with advanced disease experience demoralization, existential and spiritual distress, or loss of dignity [6, 11, 12], reflecting a loss of hope and a diminished sense of meaning [13–16]. In addition, patients and caregivers are confronted with difficult treatment decisions in palliative care planning.

Various types of psycho-oncological interventions are associated with significant, small-to-medium effects on emotional distress and quality of life [17]. However, the majority of randomized controlled psycho-oncological intervention trials for cancer patients were tested in curative settings and predominantly included women [17]. Manualized meaning-based interventions to treat the psychosocial and existential distress in cancer patients have been developed and empirically tested [18–21]. Nevertheless, the number of evaluated and effective meaning-based psychotherapeutic interventions for advanced cancer patients is still small.

Psychotherapeutic interventions for individuals with advanced disease must be short-term and flexible enough to adjust to rapidly changing needs and clinical circumstances. Only two of eight interventions described in a review of meaning-based interventions for physically ill patients by Lemay and Wilson [18] are short-term [22, 23]. Although patients with advanced disease often find it difficult to attend sessions at a fixed time in a group setting, many existing psychotherapies for cancer patients are conceptualized as group therapies [24]. Group settings in palliative care can cause additional distress if patients are faced with the death of another group member. Moreover, very few interventions focus on addressing medical treatment situations in collaboration with the medical care team [25].

Although psycho-oncological interventions have been shown to significantly reduce symptoms of anxiety and depression and enhance quality of life [17], the majority of patients with advanced cancer do not receive psychotherapeutic interventions tailored to their individual situation. To address this deficit in palliative care, Rodin and colleagues developed a brief, manualized individual psychotherapy for patients with advanced cancer: Managing Cancer and Living Meaningfully (CALM) [20, 26]. CALM aims to reduce depression and death anxiety, to strengthen communication with health care providers, and to enhance hope and meaning in life.

Given the lack of reliable data on the efficacy of psycho-oncological interventions in palliative care settings, we aim to examine the efficacy of CALM adapted for German cancer care settings compared to a non-manualized supportive psycho-oncological intervention (SPI) using a randomized control trial design (RCT).

## Methods

### Study design

**Fig. 1:**
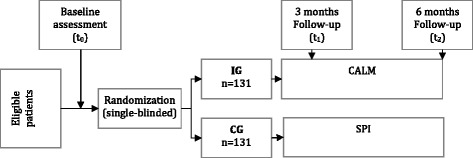
Study (RCT) design (German CALM RTC)

In this bicenter German CALM study we use a single-blinded randomized-controlled trial design with two treatment conditions: intervention group (IG) and control group (CG). Patients are assessed during screening, at baseline (t_0_), after three (t_1_) and after 6 months (t_2_). Participants are randomized between the intervention and the control arm. Patients in the intervention group receive CALM, whereas patients in the control group receive a usual non-manualized supportive psycho-oncological intervention (SPI) to the same extent (frequency and duration) as CALM (Fig. 1).

Our CALM RCT is conducted in two University Medical Centers: (a) the Outpatient Clinic for Psycho-oncology at the Department of Medical Psychology, University Medical Center Hamburg-Eppendorf and (b) the Outpatient Cancer Counseling Center at the Section of Psychosocial Oncology, Department of Medical Psychology and Medical Sociology, University Medical Center Leipzig. Our CALM RCT received ethics committee approval at both participating centers (Hamburg reference number: PV4435; Leipzig reference number: 143–14–14042014).

### Pilot phase

We conducted a pilot study prior to initiating the CALM RCT at the Outpatient Clinic for Psycho-oncology, Department of Medical Psychology, University Medical Center Hamburg-Eppendorf. We implemented this initial study phase over a 6-month period in order to test the standard operating procedures and to evaluate the feasibility of CALM in our setting. Patient recruitment followed the procedure outlined for the CALM RCT, except (a) patients were not screened for distress (DT/PHQ-9) to be considered eligible for the study and (b) there was no randomization: all participants were assigned to the intervention condition. Our pilot phase included an evaluation of each CALM therapy session and a semi-structured interview after completing at least three therapy sessions. The semi-structured interview focused on how participants experienced or evaluated (a) the overall CALM therapy; (b) each of the four CALM dimensions; (c) the therapeutic alliance, and (d) the structure and timeframe of CALM.

### Description of the experimental (CALM) and control (SPI) interventions

#### Managing Cancer and Living Meaningfully (CALM)

Patients in the IG receive the brief, individual, manualized CALM intervention [20, 26]. This semi-structured psychotherapeutic intervention was designed for patients with advanced cancer and is based on empirical research results, clinical observations and theoretical concepts of supportive-expressive, existential, and psychodynamic therapy as well as stress- and problem-solving trainings. CALM covers the following four domains:

1. *Symptom management and communication with health care providers*: Within this domain, cooperation and improvement of communication with health care providers, and help regarding medical decision-making to ensure best care and control of symptoms are addressed;
2. *Changes in self and relations with close others*: Within this domain, supporting the adjustment of self-esteem and identity to cancer-related changes, also in regard to relationships with close others, and sustaining and providing required care and support are addressed;
3. *Spirituality, sense of meaning and purpose*: Within this domain, understanding the individual meaning of suffering and dying, and evaluation of priorities and goals facing an advanced disease are addressed;
4. *Preparing for the future, sustaining hope and facing mortality*: Within this domain, acknowledgment of anticipatory fears, balancing life and death, planning advanced treatment, and preparing the process of dying are addressed.

In each CALM session, the patient decides which one of these domains and to what extent each domain is covered depending on their current concerns and supportive care needs. During a period of 6 months, patients in the IG receive up to eight sessions of individual therapy, each lasting 50 min.

#### Non-manualized supportive psycho-oncological intervention (SPI)

Patients in the CG receive a non-manualized supportive psycho-oncological intervention, which is regularly conducted by psychotherapists at both study centers, the Outpatient Cancer Counseling Center at the Section of Psychosocial Oncology, Department of Medical Psychology and Medical Sociology, University Medical Center Leipzig, and the Outpatient Clinic for Psycho-oncology at the Department of Medical Psychology, University Medical Center Hamburg-Eppendorf. The therapeutic approach is based on an integrative approach consisting of psycho-oncological counseling, information, crisis intervention as well as supportive individual therapy. As IG patients, CG patients receive up to eight sessions of individual therapy during a period of 6 months, each session lasting 50 min.

### Participants and procedure

We are recruiting patients at both study centers in Hamburg and Leipzig as well as in local university cancer care units and external cancer care facilities. Patients with advanced cancer are contacted by a study research assistant and assessed for eligibility by using the depression module of the Patient Health Questionnaire (PHQ-9) [27–29] and the Distress Thermometer (DT) [30] as screening tools. Patients fulfilling the inclusion criteria are subsequently invited to a face-to-face interview with the research assistant to receive comprehensive information about our RCT. Further inclusion and exclusion criteria are evaluated. The study research assistant conducts the Short Orientation Memory Concentration test (SOMC) [31, 32] and the depression section of the Structured Clinical Interview for DSM-IV (SCID-I) [33] to evaluate whether patients meet the criteria for a major depressive disorder. If patients meet all inclusion criteria and are willing to participate, they are randomized (single-blinded) in either the intervention or the control group. At the end of the face-to-face interview patients receive the baseline questionnaire with a prepaid envelope and are asked to return it within 2 weeks.

All patients provide written informed consent prior to participation. Principles of good research practice are strictly adhered to in this project including data and patients’ privacy protection. Patients can withdraw their informed consent at all times without having to fear any disadvantage in their medical or psychological treatment. In case patients decide not to participate in the study or to withdraw informed consent, reasons as well as basic demographic and medical characteristics (age, sex, diagnosis, date of diagnosis, educational background) are documented on a voluntary basis. These data will be used to conduct non-responder analyses and identify a potential sample bias.

### Eligibility for study participation

#### Study inclusion criteria

We include patients with (a) a malignant solid tumor who have (b) tumor stages of III or IV in accordance to the UICC classification, including advanced disease in case of sarcoma, melanoma and endocrine tumors, which implies average life expectancies between 12 and 18 months. Patients who are included in the study (c) are at least 18 years old, (d) speak German fluently, (e) score greater than or equal to nine on the PHQ-9 or/and greater than or equal to five on the Distress Thermometer (DT). It is further necessary that (f) there is no evidence of severe cognitive impairments in the patient’s records or indicated by his or her physician or oncologist, as this would interfere with the practical performance of the psychotherapeutic interventions (CALM and SPI).

#### Study exclusion criteria

During the face-to-face interview the study research assistant evaluates the following exclusion criteria: (a) deficits in communication, (b) lack of willingness or inability to attend all of the necessary therapy sessions and (c) acute suicidality (concrete suicidal thoughts and/or plans, in which case psychiatric care is provided immediately). Patients who (d) score less than 20 points on the Short Orientation-Memory-Concentration test (SOMC) or (e) show a level of less than 70 on the Karnofsky index are excluded, because life expectancy in that case is expected to be less than 6 months [34, 35]. Patients will be further excluded from the study in case they receive parallel psychotherapy. Previous experiences with psychotherapy as well as concurrent psychopharmacological treatment are documented but are no reasons to be excluded from study participation.

#### Later study exclusion criteria

Patients will be excluded from the study, if exclusion criteria occur during the intervention (e.g. cognitive impairments) or if patients show signs of acute suicidality (concrete thoughts and/or plans to commit suicide), in which case psychiatric care will be provided immediately. At the point of exclusion, study results up to this date and reasons for exclusion will be documented. These data will be further analyzed in the drop-out analysis.

### Randomization procedure

Study participants are randomly assigned to receive either CALM or SPI. The random allocation sequence (randomization list) was generated by the Institute of Medical Biometry and Epidemiology at the University Medical Center Hamburg-Eppendorf. Randomization is stratified according to sex. After a patient gives written informed consent for study participation, a study research assistant reports the patients’ initials, date of birth and sex to a blinded research assistant at the University Medical Center in Leipzig. The research assistant includes the patient in the randomization list and informs the research assistant about treatment allocation (CALM or SPI). No CALM study member and research assistant has access to the randomization list. The patient is not informed about their treatment condition.

### Assessment

#### Baseline assessment

The baseline questionnaire (t_0_) is handed to the patient by the research assistant at the end of the face-to-face interview and includes all primary and secondary outcome measures, except for the PHQ-9 depression inventory and the Distress Thermometer, which have been used as screening instruments to assess eligibility prior to the interview, and the GAD-7 anxiety inventory (Table 1).

**Table 1:** Study measures

			t_0_	t_1_	t_2_
		Screening/face-to-face interview	(baseline)	(3 months follow-up)	(6 months follow-up)
Demographic characteristics		X			
Medical and treatment-related characteristics		X		X	X
Short Orientation-Memory-Concentration test	(SOMC)	X			
Structured Clinical Interview for DSM Disorders: Major Depressive Disorder Module	(SCID–I)	X			
Distress Thermometer	(DT)	X		X	X
Depression module of the Patient Health Questionnaire	(PHQ–9)	X		X	X
Beck Depression-Inventory II	(BDI–II)			X	X
Generalized Anxiety Disorder Questionnaire	(GAD–7)	X		X	X
Demoralization Scale	(DS)		X	X	X
Brief Fatigue Inventory	(BFI)		X	X	X
Memorial Symptom Assessment Scale Short Form	(MSAS–SF)		X	X	X
Functional Assessment of Chronic Illness Therapy-Spiritual Well-Being Scale	(FACIT–SP)		X	X	X
Death and Dying Distress Scale	(DADDS)		X	X	X
Posttraumatic Growth Inventory	(PTGI)		X	X	X
The Experience in Close Relationships InventoryModified Short Term Version	(ECR–M16)		X	X	X
Quality of Life at the End of Life Cancer	(QUAL–EC)		X	X	X
Couple Communication Scale	(CCS)		X	X	X
Clinical Evaluation Questionnaire			after every therapy session		

#### Follow-up assessments

Two follow-up assessments are realized at three (t_1_) and 6 months (t_2_) following the baseline assessment. The follow-up assessments ideally correspond with the completion of the third or fourth and the sixth to eighth CALM therapy session. Participants receive the study questionnaires with a prepaid envelope and are asked to return them within 2 weeks. Those have not returned the study questionnaires within 2 weeks receive a reminder. If patients do not reply within one week after the reminder, the study research assistant will contact them by phone. The follow-up measures include the assessment of changes in demographics as well as medical and treatment-related events (e. g. new diagnoses or complications, hospitalization in the past 3 months, whether patient signed a living-will in the past 3 months) and other significant life events.

#### Evaluation of individual therapy sessions

Following each CALM or SPI therapy session, patients and therapists complete the Clinical Evaluation Questionnaire [36]. This questionnaire measures to what extent patients perceived their therapy session as helpful, and to what extent therapists felt they were supportive to the relevant aspects.

Both treatment conditions (IG and CG) end after 6 months and a maximum of eight sessions. Therefore, the last outcome assessment of the study takes place after 6 months and a maximum of eight sessions. If there is a further need for psychosocial support we offer participants the possibility to receive further treatment after study completion – either by their study therapist or a newly assigned one, according to capacity.

### Quality standards and therapists training

#### The CALM treatment manual

We translated and adapted the CALM treatment manual [37] into the German language.

#### Therapist qualifications

Our study therapists are certified psychologists with additional psycho-oncological and/or psychotherapeutic training. All study therapists are pair-randomized to the treatment conditions (IG and CG) to minimize a possible bias.

#### CALM training

CALM therapists receive a comprehensive CALM training at each study center in Hamburg or Leipzig. Therapists randomized to the control condition do not receive any additional training.

#### Supervision

To ensure the quality of the therapy under both treatment conditions, both CALM and SPI therapists receive regular supervision by experienced supervisors in separate groups. The supervisor of the IG has received the same comprehensive CALM training.

#### Tape recording

Every therapy session in both treatment conditions is audio taped. Our research team will analyze audio recordings of randomly selected therapy sessions to evaluate treatments regarding process quality.

### Main study hypotheses

We hypothesize that patients in the CALM intervention will show a significantly lower level of depression 6 months after baseline compared to patients in the control group (SPI). We further hypothesize a significant reduction in anxiety and fatigue as well as significant improvements in psychological and spiritual well-being, meaning and post-traumatic growth in the CALM intervention compared to SPI 6 months after baseline.

### Measures

Table 1 shows all study measures included in our CALM RCT. We are collecting demographic information (e. g. age, sex, marital status, education, occupational situation) through a standardized questionnaire as well as medical and treatment-related variables (e. g. cancer diagnosis, date of diagnosis, past and current medical treatments) through medical charts.

The *Short Orientation-Memory-Concentration test* (SOMC) [31, 32] is a validated culture-fair instrument for assessing orientation, memory and concentration. The SOMC scores range from 0 to 28. Scores less than 20 indicate cognitive impairments.

The *Structured Clinical Interview for DSM-IV (SCID-I)* [33] is a valid structured interview and the gold standard for assessing DSM-IV Axis I disorders. We use the 15 items representing the DSM criteria for a *major depressive disorder* in the face-to-face interview to screen whether patients meet the criteria for a major depressive disorder. The interviewer evaluates to what extent a patient’s answers meet the depression criteria from one (criterion not met) to three (criterion is fully met). To diagnose a major depressive episode at least five out of nine symptoms need to be scored with 3, one being item 1 or 2.

The German version of the *Distress Thermometer (DT)* [30] is a valid and reliable self-report instrument for screening psychological distress in cancer patients. The single-item visual analogue scale ranges from 0 (no distress) to 10 (extreme distress) to quantify the global level of distress and is accompanied by a standardized symptom checklist. In our RCT, only the single-item visual analogue scale is used. Scores equal to or higher than five indicate significant psychological distress.

The *Depression module of the Patient Health Questionnaire (PHQ-9)* [27–29] is a valid self-report screening instrument for depression. It includes nine items, which reflect the DSM-IV criteria for major depression. Items are scored on a four-point Likert scale from 0 (not at all) to 3 (nearly every day) with a total score ranging from 0 to 27. Scores up to four indicate absence of depression, scores from five to nine indicate mild depression, scores from 10 to 15 indicate moderate depression, and scores higher than 15 indicate severe levels of depression [38]. The German adaptation of the instrument shows high internal consistency with Cronbach’s α = .89 [39].

The *Beck Depression-Inventory II (BDI-II)* [40] is a valid self-report questionnaire evaluating the severity of depression. The measure consists of 21 items, which reflect the DSM-IV symptom criteria for a major depressive disorder. Items are scored on a four-point Likert-scale rated from 0 (I don’t feel particularly guilty) to 3 (I feel guilty all of the time) with a total score ranging from 0 to 63. Scores up to 13 indicate none or minimal depression, scores from 14 to 19 indicate mild depression, scores from 20 to 28 indicate moderate depression and scores higher than 29 indicate severe depression. The German BDI-II version [40] shows high internal consistency with Cronbach’s α ≥.84 for various samples [41].

The *Generalized Anxiety Disorder Questionnaire (GAD-7)* [42, 43] is a brief self-report questionnaire assessing generalized anxiety disorder. It includes seven items, which reflect the DSM-IV symptom criteria for generalized anxiety disorder. Items are scored on a four-point Likert scale ranging from 0 (not at all) to 3 (nearly every day) with a total score ranging from 0 to 21. Scores up to 4 indicate absence of anxiety, scores from 5 to 9 indicate mild anxiety, scores from 10 to 15 indicate moderate anxiety, and scores higher than15 indicate severe levels of anxiety. The German adaptation of the instrument shows high internal consistency with Cronbach’s α = .89.

The *German version of the Demoralization Scale (DS)* [44, 45] is a validated self-report instrument measuring demoralization [46]. The scale includes 24 items representing four subscales: loss of meaning and purpose, dysphoria, disheartenment and sense of failure. Items are scored on a five-point Likert scale ranging from 0 (never) to 4 (all the time) with a total score ranging from 0 to 96. Scores less than or equal to 30 indicate low demoralization whereas a score greater than 30 indicate high demoralization. The German version shows high internal consistency with Cronbach’s α = 0.84 [45].

The *Brief Fatigue Inventory (BFI)* [47, 48] is a short, validated instrument for assessing the severity of fatigue in cancer patients. After patients stated whether they felt unusually tired or fatigued during the past week, nine items measure experienced fatigue as well as its interference with certain aspects of patients’ lives within the last 24 h. Fatigue is rated on an eleven-point Likert-scale from 0 (no fatigue/does not interfere) to 10 (as bad as you can imagine/completely interferes). The mean BFI score is calculated from the nine items, with 1–4 indicating mild, 5–6 moderate and 7–10 severe fatigue. The German version shows high internal consistency with Cronbach’s α = 0.92 [48].

The *Memorial Symptom Assessment Scale (MSAS)* [49, 50] is a validated self-report instrument assessing the number of physical problems that may occur as a result of cancer or its treatment. For our study we use the list of symptoms suggested by Chang et al. [50] in their adapted version (MSAS-Short Form). The MSAS-SF assesses symptom frequency and resulting distress only. Items are scored on a five-point Likert-scale ranging from 0 (not at all) to 4 (very much). The average symptom score is calculated. The scale shows high internal consistency with Cronbach’s α = 0.87. We did not include the four psychological items of the MSAS-SF in our study because we assess psychological symptoms using other validated instruments.

The German version of the *Functional Assessment of Chronic Illness Therapy-Spiritual Well-Being Scale (FACIT-SP)* [51] is part of the FACIT measurement system. The self-report instrument measures in what way spirituality and religion contribute to the quality of life of cancer patients on the two subscales meaning/peace and faith. Items are scored on a five-point Likert-scale from 0 (not at all) to 4 (very much) with a total score ranging from 0 to 48, high scores indicating higher spiritual well-being. Subscales and the sum score show high internal consistency with Cronbach’s α = .80.

The *Death and Dying Distress Scale (DADDS)* [52, 53] is a self-report instrument assessing specific concerns of advanced cancer patients concerning insecurity about ones end of life, being a burden to others, as well as lost time and opportunities. The German adaptation includes nine instead of 15 items. Additionally, the Likert-scale was changed from a six-point to a five-point Likert-scale with mild and moderate distress put together to one label. Items can be scored from 0 (no distress) to 4 (very much distress), resulting in a sum score from 0 to 36, a higher score indicating higher distress.

The *German version of the Posttraumatic Growth Inventory (PTGI)* [54, 55] is a self-report instrument assessing personal posttraumatic growth after a traumatic event. It consists of 21 items representing four subscales: new possibilities, relating to others, appreciation of life and spiritual change. Patients are asked if their life changed because of their cancer diagnosis. Items are scored on a three-point Likert-scale ranging from 0 (not at all) to 2 (very much) with a total score ranging from 0 to 42, a high score indicating high posttraumatic growth. The German version shows high internal consistency with Cronbach’s α = 0.92 [55].

The *Experiences in Close Relationships Scale (ECR-M16)* [56, 57] is a self-report instrument assessing patients’ experiences in close romantic as well as non-romantic relationships on two subscales anxiety and avoidance. It is a shorter version of the ECR-36 [56] including 16 items due to usage in groups of highly distressed patients. Items are scored on a seven-point Likert-scale ranging from 1 (disagree) to 7 (agree) with a total score ranging from 16 to 56 on each subscale. Higher scores on one or both subscales indicate high attachment insecurity. Both subscales show high internal consistency with Cronbach’s α = 0.91 (anxiety) and α = 0.88 (avoidance) [57].

The *Quality of Life at the End of Life Cancer (QUAL-EC)* [58] is a self-report instrument assessing quality of life in patients at the end of their lives regarding their perception of a good death. The instrument includes five subscales: symptom control, relationship with health care provider, preparation for end of life and life completion. It includes 17 items, the first three items measuring symptom control and referring to three physical or emotional symptoms the patient has to name at the beginning. We used items 4–17 measuring the remaining three subscales. Items are scored on a five-point Likert-scale ranging from 1 (not at all) to 5 (completely) with a total score ranging from 14 to 70, high scores indicating high quality of life. The instrument shows high internal consistency with Cronbach’s α = 0.83.

The *Couple Communication Scale (CCS)* [59] is an online-measure for assessing communication, conflict solution and relationship satisfaction in romantic relationships and was developed as a part of the ENRICH/PREPARE program [60]. The scale is part of the Couple Checkup Scale which includes 32 items covering – depending on relationship status (dating, engaged, married) – different aspects of romantic relationships that were found to be important for a happy marriage. In accordance with G. Rodin and colleagues we only used the ten items depicting communication in romantic relationships in our study. Items can be scored from 1 (strongly disagree) to 5 (strongly agree).

The *Clinical Evaluation Questionnaire* [36] is a self-report seven-item questionnaire assessing the extent to which a therapeutic session was perceived as helpful for patients on such dimensions as being able to discuss their concerns about cancer and their treatment options and to clarify their values. The questionnaire is answered on a five-point Likert scale, ranging from 0 (not at all) to 4 (very much). Higher values indicate a higher degree of perceived benefit. If a certain aspect is not relevant, items can be rated as not applicable.

In case there was no German adaptation of the instrument available, we used state-of-the-art forward- back-translation by two independent research assistants.

### Statistical methods

#### Power calculation

To determine the sample size in a design in which two groups are compared at follow-up, controlling for baseline scores, we used the formula n = [2(Z_A_ + Z_B_)^2^ (1 − r^2^)/d^2^] + 1 suggested by Borm [61]. In a longitudinal study by Rodin et al. [25], a correlation of. 72 (based on n=137)between depression scores (BDI-II) at baseline and 6 months, as well as a SD of 7.4 at baseline were observed. Therefore, we used *r* = .70 and *SD* = 7.4 as our estimates. The goal of this RCT is to detect an effect size of Cohen’s d = .405 (d = (X_1_ − *X*_2_)/SD) [62] in depression scores between both groups (i.e. (X_1_ − *X*_2_) = d*SD = .405*7.4 = 3). Considering these factors, a minimum of 50 participants is required at t_2_.

The number of participants that need to be recruited per group at baseline is calculated by taking into account the effects of loss to follow-up and non-compliance using the formula n-_b_ = n_e_ (1/p) (1/c^2^ [63, 64]. Based on trial completion rates and compliance rates at 6 months observed in similar trials, we estimate a completion rate of 60 % (i.e. loss to follow-up rate of 40 %) and an 80 % compliance rate [65, 66]. Therefore, we need to recruit 131 participants per group, or 262 total participants at baseline. Recruitment is equally divided between Hamburg and Leipzig, resulting in a number of 66 IG patients and 66 CG patients for Hamburg and 65 patients in each group for Leipzig. The adjustment for non-compliance is conservative as it assumes that non-compliant experimental participants will respond as though they were control participants.

#### Statistical analysis

For the final analyses we will use an intention-to-treat approach (ITT) and compare patients in the assigned treatment groups. Main analyses to evaluate efficacy based on primary (depression) and secondary outcomes at 6 months follow-up (primary endpoint) will be performed using analysis of covariance (ANCOVA). In case of missing outcome data at the primary endpoint, we use the last observation carried forward (LOCF) method. Controlling for baseline test scores as well as demographic and disease-related characteristics, we hypothesize that the mean depression score in the IG will be lower than the one in the CG. Other analyses will include a mixed model approaches (to examine changes over time), regression analyses (controlling for additional factors), and exploratory analyses (of treatment effect modifiers).

## Discussion

In the last decades, existential and psychosocial needs of patients in palliative care became increasingly the focus of attention in clinical practice and research. The new World Health Organization (WHO) palliative care resolution ‘Strengthening of palliative care as a component of integrated treatment within the continuum of care’ just recently made clear that palliative care is aimed to be integrated as an essential component of health care systems worldwide [67]. In this resolution the WHO Executive Board explicitly refers to psychosocial and spiritual problems and their treatment. Despite the pronounced necessity for better psychosocial care in palliative treatment, recent studies investigating the effectiveness of psycho-oncological interventions are mostly including patients in earlier stages of the cancer disease. Most RCTs have small sample sizes and compare one active intervention against an inactive control group, no treatment, or care as usual [17].

Our CALM RCT adapted to German cancer care settings addresses the urgent need for developing, optimizing and evaluating manualized psycho-oncologic interventions for advanced cancer patients. To ensure optimal treatment for advanced patients’ specific existential and psychosocial strains it aims to close the gap of treatment options by implementing a short-term, individual and meaning-based intervention.

CALM is already under examination in a RCT in Toronto, Canada, by the research group of Prof. G. Rodin who developed CALM. Our RCT is conducted in close cooperation with the Canadian research group, which ensures research quality and helps us to be prepared for possible risks and obstacles during the trial. Currently, studies examining CALM are also conducted in Italy and the United Kingdom, which will lead to a growing international application of the intervention as a psychological dimension of early palliative care. As one of the few psycho-oncological interventions dealing with these issues, our study will contribute important statistical evidence on whether CALM can reduce depression and existential distress in a German sample of advanced and highly distressed cancer patients.

## Abbreviations

CALM: Managing Cancer and Living Meaningfully
CAU: Care as usual
CG: Control group
DT: Distress thermometer
IG: Intervention group
ITT: Intention-to-treat
LOCF: Last observation carried forward
RCT: Randomized controlled trial
SPI: Non-manualized supportive psycho-oncological intervention
